# 3′-(4-Chloro­benzo­yl)-4′-(4-chloro­phen­yl)-1′-methyl­spiro­[indoline-3,2′-pyrrolidin]-2-one

**DOI:** 10.1107/S1600536811044618

**Published:** 2011-10-29

**Authors:** T. Srinivasan, S. Suhitha, S. Purushothaman, R. Raghunathan, D. Velmurugan

**Affiliations:** a Centre of Advanced Study in Crystallography and Biophysics, University of Madras, Guindy Campus, Chennai 600 025, India; b Department of Organic Chemistry, University of Madras, Guindy Campus, Chennai 600 025, India

## Abstract

In the title compound, C_25_H_20_Cl_2_N_2_O_2_, the pyrrolidine ring adopts an envelope conformation and the best plane through the five ring atoms makes a dihedral angle of 87.03 (8)° with the indoline ring. Mol­ecules are connected by pairs of N—H⋯O hydrogen bonds into centrosymmetric dimers with an *R*_2_^2^(8) graph-set ring motif. C—H⋯O hydrogen bonds stabilize the crystal structure.

## Related literature

For substituted pyrrolidine compounds, see: Coldham & Hufton (2005 ▶). For graph-set notation of hydrogen bonds, see: Bernstein *et al.* (1995 ▶).
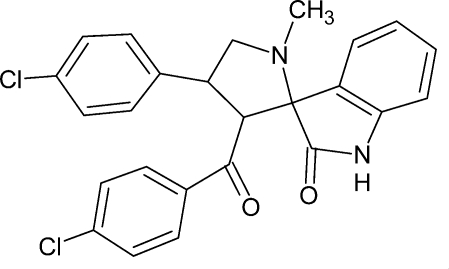


## Experimental

### 

#### Crystal data

C_25_H_20_Cl_2_N_2_O_2_
*M* *_r_* = 451.33
Monoclinic, 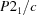

*a* = 11.4139 (2) Å
*b* = 11.6957 (2) Å
*c* = 16.5262 (2) Å
β = 102.037 (1)°
*V* = 2157.64 (6) Å^3^
*Z* = 4
Mo *K*α radiation
μ = 0.33 mm^−1^
*T* = 293 K
0.20 × 0.20 × 0.20 mm

#### Data collection

Bruker SMART APEXII area-detector diffractometer
20528 measured reflections
5426 independent reflections
3812 reflections with *I* > 2σ(*I*)
*R*_int_ = 0.026

#### Refinement

*R*[*F* ^2^ > 2σ(*F* ^2^)] = 0.041
*wR*(*F* ^2^) = 0.114
*S* = 1.03
5426 reflections
293 parameters
H atoms treated by a mixture of independent and constrained refinement
Δρ_max_ = 0.33 e Å^−3^
Δρ_min_ = −0.44 e Å^−3^

### 

Data collection: *APEX2* (Bruker, 2008 ▶); cell refinement: *SAINT* (Bruker, 2008 ▶); data reduction: *SAINT*; program(s) used to solve structure: *SHELXS97* (Sheldrick, 2008 ▶); program(s) used to refine structure: *SHELXL97* (Sheldrick, 2008 ▶); molecular graphics: *ORTEP-3* (Farrugia, 1997 ▶); software used to prepare material for publication: *SHELXL97* and *PLATON* (Spek, 2009 ▶).

## Supplementary Material

Crystal structure: contains datablock(s) global, I. DOI: 10.1107/S1600536811044618/bt5687sup1.cif

Structure factors: contains datablock(s) I. DOI: 10.1107/S1600536811044618/bt5687Isup2.hkl

Supplementary material file. DOI: 10.1107/S1600536811044618/bt5687Isup3.cml

Additional supplementary materials:  crystallographic information; 3D view; checkCIF report

## Figures and Tables

**Table 1:** Hydrogen-bond geometry (Å, °)

*D*—H⋯*A*	*D*—H	H⋯*A*	*D*⋯*A*	*D*—H⋯*A*
N2—H2*A*⋯O2^i^	0.85 (2)	2.06 (2)	2.876 (2)	160
C24—H24⋯O1^ii^	0.93	2.42	3.104 (2)	130

Symmetry codes: (i) 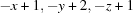; (ii) 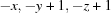.

**Fig. 1.**
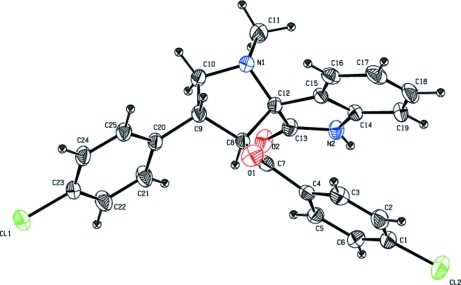
The molecular structure of the title compound, showing displacement ellipsoids drawn at the 30% probability level. H atoms are presented as a small spheres of arbitrary radius.

**Fig. 2.**
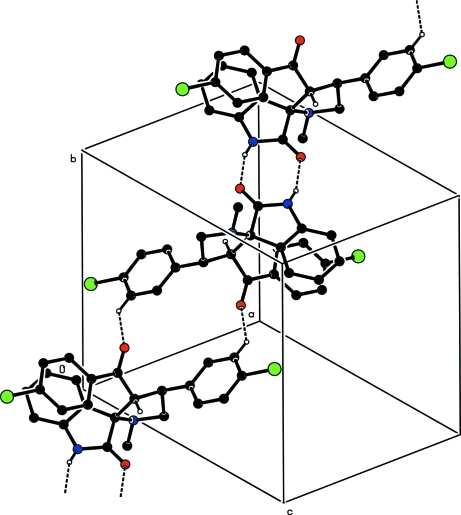
The crystal packing of the title compound viewed down b axis, showing the hydrogen bonds resulting in R22(16) and R22(8) graph-set ring motifs; H-atoms not involved in H-bonds have been excluded for clarity.

## supplementary crystallographic information

### Comment

Substituted pyrrolidine compounds are an important class of heterocyclic compounds with wide spread applications to the synthesis of biologically active compounds and natural products (Coldham & Hufton, 2005).

The indoline ring is essentially planar with a maximum deviation of 0.0594 (16)Å for atom C12. The oxygen atom O2 deviates with the value of 0.0566 (13)Å from the indoline ring. The phenyl ring of chlorophenyl group makes a dihedral angle of 79.68 (9)° and 20.47 (7)° with the pyrollidin ring and indoline ring system. The phenyl ring of chlorobenzaldehyde group makes a dihedral angle of 71.39 (9)° and 35.17 (8)° with the pyrrolidin ring and indoline ring system, respectively.

The pyrrolidin ring adopts an *envelope* conformation. The pyrrolidin ring makes a dihedral angle of 87.03 (8)° with the indoline ring system. The crystal structure is stabilized by C—H···O and N—H···O hydrogen bonds resulting in R^2^_2_(16) and R^2^_2_(8) graph-set ring motifs (Bernstein *et al.*, 1995).

### Experimental

A solution of (E)-1,3-bis(4-chlorophenyl)prop-2-en-1-one(2 mmol), isatin (1 eq.) and sarcosine( 1 eq.) was refluxed in dry toluene for 8 hrs at 110°C using Dean-Stark apparatus.After the completion of reaction as indicated by TLC,toluene was evaporated under reduced pressure.The crude product was purified by column chromatography using hexane: EtOAc (8:2) as eluent.

### Refinement

The hydrogen atoms were placed in calculated positions with C—H = 0.93Å to 1.00Å  and refined in the riding model with fixed isotropic displacement parameters: *U*_iso_(H) = 1.5*U*_eq_(C) for methyl group and *U*_iso_(H) = 1.2*U*_eq_(C) for other H atoms.

### Crystal data

**Table:**

C_25_H_20_Cl_2_N_2_O_2_	*F*(000) = 936
*M**_r_* = 451.33	*D*_x_ = 1.389 Mg m^−^^3^
Monoclinic, *P*2_1_/*c*	Mo *K*α radiation, λ = 0.71073 Å
Hall symbol: -P 2ybc	Cell parameters from 5426 reflections
*a* = 11.4139 (2) Å	θ = 1.8–28.4°
*b* = 11.6957 (2) Å	µ = 0.33 mm^−^^1^
*c* = 16.5262 (2) Å	*T* = 293 K
β = 102.037 (1)°	Block, colourless
*V* = 2157.64 (6) Å^3^	0.20 × 0.20 × 0.20 mm
*Z* = 4	

### Data collection

**Table:**

Bruker SMART APEXII area-detector diffractometer	3812 reflections with *I* > 2σ(*I*)
Radiation source: fine-focus sealed tube	*R*_int_ = 0.026
graphite	θ_max_ = 28.4°, θ_min_ = 1.8°
ω and φ scans	*h* = −15→13
20528 measured reflections	*k* = −15→15
5426 independent reflections	*l* = −22→22

### Refinement

**Table:**

Refinement on *F*^2^	Primary atom site location: structure-invariant direct methods
Least-squares matrix: full	Secondary atom site location: difference Fourier map
*R*[*F*^2^ > 2σ(*F*^2^)] = 0.041	Hydrogen site location: inferred from neighbouring sites
*wR*(*F*^2^) = 0.114	H atoms treated by a mixture of independent and constrained refinement
*S* = 1.03	*w* = 1/[σ^2^(*F*_o_^2^) + (0.0497*P*)^2^ + 0.5071*P*] where *P* = (*F*_o_^2^ + 2*F*_c_^2^)/3
5426 reflections	(Δ/σ)_max_ < 0.001
293 parameters	Δρ_max_ = 0.33 e Å^−^^3^
0 restraints	Δρ_min_ = −0.44 e Å^−^^3^

### Special details

**Table:**

Geometry. All esds (except the esd in the dihedral angle between two l.s. planes) are estimated using the full covariance matrix. The cell esds are taken into account individually in the estimation of esds in distances, angles and torsion angles; correlations between esds in cell parameters are only used when they are defined by crystal symmetry. An approximate (isotropic) treatment of cell esds is used for estimating esds involving l.s. planes.
Refinement. Refinement of F^2^ against ALL reflections. The weighted R-factor wR and goodness of fit S are based on F^2^, conventional R-factors R are based on F, with F set to zero for negative F^2^. The threshold expression of F^2^ > 2sigma(F^2^) is used only for calculating R-factors(gt) etc. and is not relevant to the choice of reflections for refinement. R-factors based on F^2^ are statistically about twice as large as those based on F, and R- factors based on ALL data will be even larger.

### Fractional atomic coordinates and isotropic or equivalent isotropic displacement parameters (Å^2^)

**Table:**

	*x*	*y*	*z*	*U*_iso_*/*U*_eq_	
Cl1	−0.14204 (5)	0.56050 (5)	0.16763 (3)	0.06775 (17)	
Cl2	0.85262 (5)	0.60950 (6)	0.62529 (4)	0.0862 (2)	
C9	0.11880 (14)	0.73473 (15)	0.50025 (9)	0.0400 (4)	
N2	0.48172 (13)	0.92400 (12)	0.60195 (9)	0.0433 (3)	
C15	0.37099 (14)	0.80302 (13)	0.66407 (8)	0.0367 (3)	
C7	0.31713 (14)	0.62491 (14)	0.54566 (9)	0.0394 (3)	
O2	0.34978 (11)	0.96763 (11)	0.48075 (8)	0.0564 (3)	
N1	0.17789 (11)	0.88494 (12)	0.59138 (8)	0.0420 (3)	
C8	0.25585 (13)	0.73727 (14)	0.51710 (9)	0.0359 (3)	
C12	0.29245 (13)	0.83649 (13)	0.58229 (8)	0.0350 (3)	
C20	0.05641 (13)	0.69085 (15)	0.41599 (9)	0.0407 (4)	
C23	−0.06461 (15)	0.61146 (16)	0.26300 (10)	0.0472 (4)	
O1	0.25970 (12)	0.54417 (11)	0.56163 (9)	0.0602 (3)	
C4	0.45034 (14)	0.61803 (13)	0.55866 (9)	0.0381 (3)	
C13	0.37572 (14)	0.91885 (14)	0.54743 (9)	0.0396 (3)	
C14	0.48245 (14)	0.85308 (14)	0.67070 (9)	0.0402 (3)	
C10	0.08849 (14)	0.85794 (16)	0.51710 (10)	0.0457 (4)	
H10A	0.0963	0.9076	0.4715	0.055*	
H10B	0.0079	0.8643	0.5270	0.055*	
C5	0.51767 (14)	0.68787 (15)	0.51797 (9)	0.0429 (4)	
H5	0.4790	0.7376	0.4771	0.052*	
C25	−0.04115 (14)	0.61909 (16)	0.40920 (10)	0.0459 (4)	
H25	−0.0664	0.5969	0.4568	0.055*	
C1	0.69779 (16)	0.61090 (17)	0.59772 (11)	0.0533 (4)	
C24	−0.10233 (15)	0.57939 (16)	0.33323 (10)	0.0484 (4)	
H24	−0.1682	0.5315	0.3298	0.058*	
C6	0.64165 (15)	0.68453 (17)	0.53744 (11)	0.0506 (4)	
H6	0.6862	0.7316	0.5100	0.061*	
C22	0.03220 (16)	0.68206 (19)	0.26713 (10)	0.0593 (5)	
H22	0.0576	0.7029	0.2193	0.071*	
C16	0.34817 (17)	0.73153 (15)	0.72517 (9)	0.0465 (4)	
H16	0.2726	0.6999	0.7218	0.056*	
C2	0.63333 (18)	0.53729 (17)	0.63664 (11)	0.0570 (5)	
H2	0.6726	0.4854	0.6757	0.068*	
C17	0.4410 (2)	0.70793 (18)	0.79193 (10)	0.0600 (5)	
H17	0.4278	0.6597	0.8339	0.072*	
C3	0.50996 (17)	0.54119 (15)	0.61720 (10)	0.0500 (4)	
H3	0.4661	0.4918	0.6436	0.060*	
C21	0.09178 (16)	0.72204 (18)	0.34360 (10)	0.0563 (5)	
H21	0.1569	0.7708	0.3465	0.068*	
C19	0.57585 (17)	0.82942 (18)	0.73636 (10)	0.0556 (5)	
H19	0.6512	0.8619	0.7400	0.067*	
C18	0.5529 (2)	0.75563 (19)	0.79644 (11)	0.0658 (6)	
H18	0.6143	0.7376	0.8411	0.079*	
C11	0.18210 (18)	1.00343 (17)	0.61905 (12)	0.0593 (5)	
H11A	0.1973	1.0524	0.5758	0.089*	
H11B	0.2450	1.0123	0.6672	0.089*	
H11C	0.1068	1.0236	0.6324	0.089*	
H2A	0.5428 (19)	0.9548 (17)	0.5892 (12)	0.059 (6)*	
H8	0.2804 (14)	0.7597 (13)	0.4670 (10)	0.037 (4)*	
H9	0.0928 (14)	0.6848 (14)	0.5430 (10)	0.039 (4)*	

### Atomic displacement parameters (Å^2^)

**Table:**

	*U*^11^	*U*^22^	*U*^33^	*U*^12^	*U*^13^	*U*^23^
Cl1	0.0563 (3)	0.0957 (4)	0.0465 (2)	−0.0108 (3)	0.0000 (2)	−0.0216 (2)
Cl2	0.0442 (3)	0.1103 (5)	0.0945 (4)	0.0194 (3)	−0.0074 (3)	−0.0141 (4)
C9	0.0320 (8)	0.0555 (10)	0.0323 (7)	−0.0082 (7)	0.0064 (6)	0.0019 (7)
N2	0.0359 (7)	0.0450 (8)	0.0466 (7)	−0.0103 (6)	0.0036 (6)	0.0004 (6)
C15	0.0392 (8)	0.0399 (8)	0.0291 (6)	0.0034 (7)	0.0031 (6)	−0.0034 (6)
C7	0.0436 (9)	0.0440 (9)	0.0317 (7)	−0.0064 (7)	0.0103 (6)	−0.0045 (6)
O2	0.0453 (7)	0.0658 (8)	0.0553 (7)	−0.0057 (6)	0.0045 (6)	0.0267 (6)
N1	0.0338 (7)	0.0526 (8)	0.0384 (7)	0.0027 (6)	0.0051 (5)	−0.0035 (6)
C8	0.0311 (7)	0.0481 (9)	0.0286 (6)	−0.0063 (6)	0.0061 (6)	0.0009 (6)
C12	0.0321 (8)	0.0400 (8)	0.0323 (7)	−0.0030 (6)	0.0052 (6)	0.0020 (6)
C20	0.0323 (8)	0.0534 (9)	0.0351 (7)	−0.0051 (7)	0.0042 (6)	0.0016 (7)
C23	0.0367 (9)	0.0630 (11)	0.0385 (8)	−0.0009 (8)	−0.0002 (7)	−0.0070 (8)
O1	0.0583 (8)	0.0485 (7)	0.0773 (9)	−0.0123 (6)	0.0220 (7)	0.0039 (6)
C4	0.0425 (9)	0.0401 (8)	0.0315 (7)	0.0011 (7)	0.0072 (6)	−0.0053 (6)
C13	0.0355 (8)	0.0408 (8)	0.0416 (8)	−0.0027 (7)	0.0057 (6)	0.0040 (6)
C14	0.0388 (8)	0.0441 (9)	0.0353 (7)	0.0022 (7)	0.0022 (6)	−0.0070 (6)
C10	0.0312 (8)	0.0642 (11)	0.0405 (8)	0.0012 (7)	0.0049 (6)	0.0004 (7)
C5	0.0403 (9)	0.0534 (10)	0.0358 (7)	0.0054 (7)	0.0095 (6)	0.0036 (7)
C25	0.0345 (8)	0.0643 (11)	0.0385 (8)	−0.0076 (8)	0.0069 (6)	0.0054 (7)
C1	0.0427 (10)	0.0620 (12)	0.0509 (9)	0.0114 (9)	0.0000 (8)	−0.0145 (9)
C24	0.0331 (8)	0.0616 (11)	0.0479 (9)	−0.0104 (8)	0.0022 (7)	−0.0002 (8)
C6	0.0394 (9)	0.0638 (11)	0.0500 (9)	0.0034 (8)	0.0121 (7)	−0.0015 (8)
C22	0.0511 (11)	0.0909 (15)	0.0356 (8)	−0.0186 (10)	0.0085 (7)	0.0009 (9)
C16	0.0585 (11)	0.0490 (9)	0.0337 (7)	0.0051 (8)	0.0131 (7)	0.0009 (7)
C2	0.0621 (12)	0.0564 (11)	0.0459 (9)	0.0184 (9)	−0.0036 (8)	−0.0010 (8)
C17	0.0856 (15)	0.0617 (12)	0.0319 (8)	0.0163 (11)	0.0102 (9)	0.0049 (8)
C3	0.0617 (12)	0.0435 (10)	0.0450 (9)	0.0041 (8)	0.0115 (8)	0.0025 (7)
C21	0.0464 (10)	0.0823 (14)	0.0395 (8)	−0.0263 (9)	0.0070 (7)	0.0000 (9)
C19	0.0455 (10)	0.0719 (13)	0.0424 (9)	0.0046 (9)	−0.0070 (7)	−0.0127 (9)
C18	0.0732 (14)	0.0810 (15)	0.0340 (8)	0.0263 (12)	−0.0098 (9)	−0.0059 (9)
C11	0.0514 (11)	0.0609 (12)	0.0614 (11)	0.0097 (9)	0.0019 (9)	−0.0120 (9)

### Geometric parameters (Å, °)

**Table:**

Cl1—C23	1.7433 (16)	C14—C19	1.381 (2)
Cl2—C1	1.7306 (19)	C10—H10A	0.9700
C9—C20	1.515 (2)	C10—H10B	0.9700
C9—C10	1.521 (2)	C5—C6	1.385 (2)
C9—C8	1.531 (2)	C5—H5	0.9300
C9—H9	1.007 (16)	C25—C24	1.383 (2)
N2—C13	1.351 (2)	C25—H25	0.9300
N2—C14	1.405 (2)	C1—C6	1.371 (3)
N2—H2A	0.85 (2)	C1—C2	1.376 (3)
C15—C16	1.377 (2)	C24—H24	0.9300
C15—C14	1.384 (2)	C6—H6	0.9300
C15—C12	1.5093 (19)	C22—C21	1.386 (2)
C7—O1	1.2096 (19)	C22—H22	0.9300
C7—C4	1.493 (2)	C16—C17	1.389 (3)
C7—C8	1.517 (2)	C16—H16	0.9300
O2—C13	1.2213 (19)	C2—C3	1.378 (3)
N1—C11	1.457 (2)	C2—H2	0.9300
N1—C10	1.458 (2)	C17—C18	1.381 (3)
N1—C12	1.4612 (19)	C17—H17	0.9300
C8—C12	1.579 (2)	C3—H3	0.9300
C8—H8	0.964 (15)	C21—H21	0.9300
C12—C13	1.546 (2)	C19—C18	1.381 (3)
C20—C25	1.380 (2)	C19—H19	0.9300
C20—C21	1.389 (2)	C18—H18	0.9300
C23—C22	1.370 (2)	C11—H11A	0.9600
C23—C24	1.372 (2)	C11—H11B	0.9600
C4—C5	1.388 (2)	C11—H11C	0.9600
C4—C3	1.390 (2)		
			
C20—C9—C10	114.07 (14)	N1—C10—H10B	111.3
C20—C9—C8	116.12 (12)	C9—C10—H10B	111.3
C10—C9—C8	102.19 (13)	H10A—C10—H10B	109.2
C20—C9—H9	107.3 (9)	C6—C5—C4	120.88 (15)
C10—C9—H9	108.0 (9)	C6—C5—H5	119.6
C8—C9—H9	108.8 (9)	C4—C5—H5	119.6
C13—N2—C14	111.50 (14)	C20—C25—C24	121.60 (15)
C13—N2—H2A	121.4 (13)	C20—C25—H25	119.2
C14—N2—H2A	125.7 (14)	C24—C25—H25	119.2
C16—C15—C14	120.67 (14)	C6—C1—C2	121.22 (17)
C16—C15—C12	130.26 (15)	C6—C1—Cl2	119.63 (16)
C14—C15—C12	109.01 (13)	C2—C1—Cl2	119.16 (15)
O1—C7—C4	120.57 (15)	C23—C24—C25	119.21 (15)
O1—C7—C8	120.56 (15)	C23—C24—H24	120.4
C4—C7—C8	118.76 (13)	C25—C24—H24	120.4
C11—N1—C10	116.21 (14)	C1—C6—C5	119.13 (17)
C11—N1—C12	115.43 (13)	C1—C6—H6	120.4
C10—N1—C12	108.29 (12)	C5—C6—H6	120.4
C7—C8—C9	115.29 (13)	C23—C22—C21	119.11 (16)
C7—C8—C12	112.65 (12)	C23—C22—H22	120.4
C9—C8—C12	104.67 (12)	C21—C22—H22	120.4
C7—C8—H8	107.7 (9)	C15—C16—C17	118.25 (18)
C9—C8—H8	108.6 (9)	C15—C16—H16	120.9
C12—C8—H8	107.7 (9)	C17—C16—H16	120.9
N1—C12—C15	112.69 (12)	C1—C2—C3	119.36 (17)
N1—C12—C13	115.44 (13)	C1—C2—H2	120.3
C15—C12—C13	101.46 (12)	C3—C2—H2	120.3
N1—C12—C8	103.88 (11)	C18—C17—C16	120.42 (18)
C15—C12—C8	116.32 (13)	C18—C17—H17	119.8
C13—C12—C8	107.41 (11)	C16—C17—H17	119.8
C25—C20—C21	117.66 (14)	C2—C3—C4	120.78 (17)
C25—C20—C9	119.82 (13)	C2—C3—H3	119.6
C21—C20—C9	122.51 (14)	C4—C3—H3	119.6
C22—C23—C24	120.99 (15)	C22—C21—C20	121.43 (16)
C22—C23—Cl1	120.19 (13)	C22—C21—H21	119.3
C24—C23—Cl1	118.82 (13)	C20—C21—H21	119.3
C5—C4—C3	118.53 (15)	C18—C19—C14	117.30 (18)
C5—C4—C7	123.30 (14)	C18—C19—H19	121.3
C3—C4—C7	118.11 (15)	C14—C19—H19	121.3
O2—C13—N2	126.58 (15)	C17—C18—C19	121.72 (17)
O2—C13—C12	125.09 (14)	C17—C18—H18	119.1
N2—C13—C12	108.29 (13)	C19—C18—H18	119.1
C19—C14—C15	121.57 (16)	N1—C11—H11A	109.5
C19—C14—N2	128.82 (16)	N1—C11—H11B	109.5
C15—C14—N2	109.59 (13)	H11A—C11—H11B	109.5
N1—C10—C9	102.32 (13)	N1—C11—H11C	109.5
N1—C10—H10A	111.3	H11A—C11—H11C	109.5
C9—C10—H10A	111.3	H11B—C11—H11C	109.5
			
O1—C7—C8—C9	−7.3 (2)	C15—C12—C13—N2	1.53 (16)
C4—C7—C8—C9	176.42 (12)	C8—C12—C13—N2	−121.00 (14)
O1—C7—C8—C12	112.75 (16)	C16—C15—C14—C19	3.0 (2)
C4—C7—C8—C12	−63.54 (16)	C12—C15—C14—C19	−174.50 (15)
C20—C9—C8—C7	−83.09 (17)	C16—C15—C14—N2	−178.46 (14)
C10—C9—C8—C7	152.12 (12)	C12—C15—C14—N2	4.06 (17)
C20—C9—C8—C12	152.59 (13)	C13—N2—C14—C19	175.32 (17)
C10—C9—C8—C12	27.79 (14)	C13—N2—C14—C15	−3.10 (19)
C11—N1—C12—C15	77.91 (17)	C11—N1—C10—C9	173.24 (14)
C10—N1—C12—C15	−149.85 (13)	C12—N1—C10—C9	41.42 (15)
C11—N1—C12—C13	−38.02 (18)	C20—C9—C10—N1	−167.97 (12)
C10—N1—C12—C13	94.22 (15)	C8—C9—C10—N1	−41.82 (14)
C11—N1—C12—C8	−155.33 (13)	C3—C4—C5—C6	−2.5 (2)
C10—N1—C12—C8	−23.09 (15)	C7—C4—C5—C6	174.50 (15)
C16—C15—C12—N1	55.4 (2)	C21—C20—C25—C24	−0.2 (3)
C14—C15—C12—N1	−127.39 (14)	C9—C20—C25—C24	178.42 (16)
C16—C15—C12—C13	179.49 (16)	C22—C23—C24—C25	−0.1 (3)
C14—C15—C12—C13	−3.36 (16)	Cl1—C23—C24—C25	179.77 (14)
C16—C15—C12—C8	−64.4 (2)	C20—C25—C24—C23	0.5 (3)
C14—C15—C12—C8	112.81 (14)	C2—C1—C6—C5	2.7 (3)
C7—C8—C12—N1	−130.00 (12)	Cl2—C1—C6—C5	−177.68 (13)
C9—C8—C12—N1	−4.01 (14)	C4—C5—C6—C1	0.1 (3)
C7—C8—C12—C15	−5.56 (17)	C24—C23—C22—C21	−0.5 (3)
C9—C8—C12—C15	120.43 (14)	Cl1—C23—C22—C21	179.58 (17)
C7—C8—C12—C13	107.23 (14)	C14—C15—C16—C17	−2.3 (2)
C9—C8—C12—C13	−126.78 (12)	C12—C15—C16—C17	174.61 (16)
C10—C9—C20—C25	−102.97 (18)	C6—C1—C2—C3	−2.9 (3)
C8—C9—C20—C25	138.57 (16)	Cl2—C1—C2—C3	177.48 (14)
C10—C9—C20—C21	75.5 (2)	C15—C16—C17—C18	0.2 (3)
C8—C9—C20—C21	−42.9 (2)	C1—C2—C3—C4	0.3 (3)
O1—C7—C4—C5	157.76 (16)	C5—C4—C3—C2	2.3 (2)
C8—C7—C4—C5	−25.9 (2)	C7—C4—C3—C2	−174.86 (15)
O1—C7—C4—C3	−25.2 (2)	C23—C22—C21—C20	0.9 (3)
C8—C7—C4—C3	151.11 (14)	C25—C20—C21—C22	−0.5 (3)
C14—N2—C13—O2	−177.25 (17)	C9—C20—C21—C22	−179.06 (19)
C14—N2—C13—C12	0.80 (18)	C15—C14—C19—C18	−1.5 (3)
N1—C12—C13—O2	−58.2 (2)	N2—C14—C19—C18	−179.75 (17)
C15—C12—C13—O2	179.62 (16)	C16—C17—C18—C19	1.3 (3)
C8—C12—C13—O2	57.1 (2)	C14—C19—C18—C17	−0.6 (3)
N1—C12—C13—N2	123.69 (14)		

### Hydrogen-bond geometry (Å, °)

**Table:**

*D*—H···*A*	*D*—H	H···*A*	*D*···*A*	*D*—H···*A*
N2—H2A···O2^i^	0.85 (2)	2.06 (2)	2.876 (2)	160
C24—H24···O1^ii^	0.93	2.42	3.104 (2)	130

Symmetry codes: (i) −*x*+1, −*y*+2, −*z*+1; (ii) −*x*, −*y*+1, −*z*+1.

## References

[bb1] Bernstein, J., Davis, R. E., Shimoni, L. & Chang, N.-L. (1995). Angew. Chem. Int. Ed. Engl. 34, 1555–1573.

[bb2] Bruker (2008). *APEX2* and *SAINT* Bruker AXS Inc., Madison, Wisconsin, USA.

[bb3] Coldham, I. & Hufton, R. (2005). *Chem. Rev.* **105**, 2765–2810. 10.1021/cr040004c 16011324

[bb4] Farrugia, L. J. (1997). *J. Appl. Cryst.* **30**, 565.

[bb5] Sheldrick, G. M. (2008). *Acta Cryst.* A**64**, 112–122. 10.1107/S0108767307043930 18156677

[bb6] Spek, A. L. (2009). *Acta Cryst.* D**65**, 148–155. 10.1107/S090744490804362X PMC2631630 19171970

